# Human Pluripotent Stem Cells-Based Therapies for Neurodegenerative Diseases: Current Status and Challenges

**DOI:** 10.3390/cells9112517

**Published:** 2020-11-20

**Authors:** Elizabeth Ford, Jodie Pearlman, Travis Ruan, John Manion, Matthew Waller, Gregory G. Neely, Leslie Caron

**Affiliations:** 1 Dr. John and Anne Chong Lab for Functional Genomics, Charles Perkins Centre, Centenary Institute, University of Sydney, Camperdown, NSW 2006, Australia; elizabeth.ford@bath.edu (E.F.); jodie.pearlman@bath.edu (J.P.); travis.ruan@sydney.edu.au (T.R.); John.Manion@childrens.harvard.edu (J.M.); matthew.waller@sydney.edu.au (M.W.); greg.neely@sydney.edu.au (G.G.N.); 2 School of Life and Environmental Sciences, University of Sydney, Camperdown, NSW 2006, Australia; 3 Department of Biology and Biochemistry, University of Bath, Bath BA2 7AY, UK; 4 Department of Urology, Boston Children’s Hospital, Boston, MA 02115, USA; 5 Departments of Surgery and Microbiology, Harvard Medical School, Boston, MA 02115, USA

**Keywords:** neurodegenerative diseases, human pluripotent stem cells, cell therapy, regenerative medicine

## Abstract

Neurodegenerative diseases are characterized by irreversible cell damage, loss of neuronal cells and limited regeneration potential of the adult nervous system. Pluripotent stem cells are capable of differentiating into the multitude of cell types that compose the central and peripheral nervous systems and so have become the major focus of cell replacement therapies for the treatment of neurological disorders. Human embryonic stem cell (hESC) and human induced pluripotent stem cell (hiPSC)-derived cells have both been extensively studied as cell therapies in a wide range of neurodegenerative disease models in rodents and non-human primates, including Parkinson’s disease, stroke, epilepsy, spinal cord injury, Alzheimer’s disease, multiple sclerosis and pain. In this review, we discuss the latest progress made with stem cell therapies targeting these pathologies. We also evaluate the challenges in clinical application of human pluripotent stem cell (hPSC)-based therapies including risk of oncogenesis and tumor formation, immune rejection and difficulty in regeneration of the heterogeneous cell types composing the central nervous system.

## 1. Introduction

Stem cell biology is indisputably a fast-moving field of research and over the past few years human pluripotent stem cells (hPSC)-based therapies have progressed into early clinical trials, with several patients now receiving hPSC-derived cells. Transplantations of retinal pigmented epithelium (RPE) derived from human embryonic stem cells (hESC) [1,2,3] and human induced pluripotent stem cells (hiPSC) [4] have been tested in patients with age-related macular degeneration (AMD), a leading cause of blindness among the older population [5] and have shown promising results in stalling disease progression. HiPSC-based therapy for the treatment of Parkinson’s disease (PD) entered clinical trials in 2018, almost 30 years after the first transplants of human fetal material revealed the potential for dopaminergic cell transplants [6,7]. In August 2019, a patient in Japan received the first corneal transplant made from hiPSC. Although it is still too early to ascertain the efficacy of the procedure, the patient’s vision has reportedly improved considerably since the transplantation. Clinical trials using hiPSC to treat spinal cord injury are also about to start in Japan [8]. The remarkable clinical advance that these first-in-human hPSC- based therapies represent has generated considerable excitement. However, ethical and safety concerns associated with hPSC transplantation persist and clinical application of stem cells remains a daunting task. Although a large repertoire of hPSC-derived cell types (>40) can be generated in vitro, each has its own challenges to overcome to be suitable for cell therapy. Regenerative medicine should therefore address pathologies or conditions with unmet medical needs only. Nonetheless, stem cell therapy may represent the most promising approach for a large number of degenerative diseases given the limited capacity of intrinsic tissue regeneration.

Over the past few decades, stem cell therapies have advanced further towards the clinic to treat damaged tissues and various degenerative diseases, especially those affecting the nervous system. Neurodegenerative diseases are frequently characterized by the irreversible functional impairment and/or loss of cells and limited regeneration of the adult nervous system. Cell replacement, therefore, represents a critical strategy for the effective treatment of neurodegenerative diseases. HPSC can be differentiated into many kinds of neuronal cells, from neural progenitors to specialized mature neurons, astrocytes or oligodendrocytes. All have great potential in treating neuropathies such as PD, multiple sclerosis (MS) or spinal cord injury (SCI). Recently, two studies have reported oncogenic mutations in hiPSC [4] and hESC [9] and raised the importance of designing new strategies for safe and efficient production and functional validation of hPSC-derived cells that can be used for transplantation. However, there are still debates as to whether stem cell therapy is feasible, safe and efficient or just conceptual. Considering the rising interest in stem cell-based therapies, a better understanding of their implication in the treatment of neurodegenerative diseases is critical for the design of appropriate strategies. It is therefore both a timely and a necessary question to review the current state of hPSC-based therapies and their relevance in nervous system regeneration. In this review, we discuss the progress made with cellular therapies, where cells derived from hPSC are directly delivered in vivo, in the context of central nervous system (CNS) pathologies. Lastly, we review the challenges of working with hPSC and discuss the hurdles that must be overcome to translate these promising approaches to the clinic for the treatment of neurodegenerative diseases. 

## 2. Main Text

### 2.1. Parkinson’s Disease

Parkinson’s disease (PD) is a progressive neurodegenerative disorder, characterized by a progressive loss of dopaminergic neurons (DANs) within the substantia nigra, subsequently causing bradykinesia, tremor and other debilitating symptoms such as cognitive decline. Therapies that increase dopamine (DA) level in the brain, including the DA precursor L-DOPA (levodopa), and deep brain stimulation are currently the gold standard treatment for PD [10]. Administration of L-DOPA can have dramatic effects on bradykinesia and is often an effective initial treatment to improve the quality of life of some patients. However, as the pathology progresses a decreasing number of dopaminergic neurons are able to convert L-DOPA and fewer synapses are able to release dopamine. Subsequently the treatment loses efficacy[11,12]. Hence, cell replacement of lost DANs for PD has long been pursued and has profound clinical interest. 

**Figure 1:**
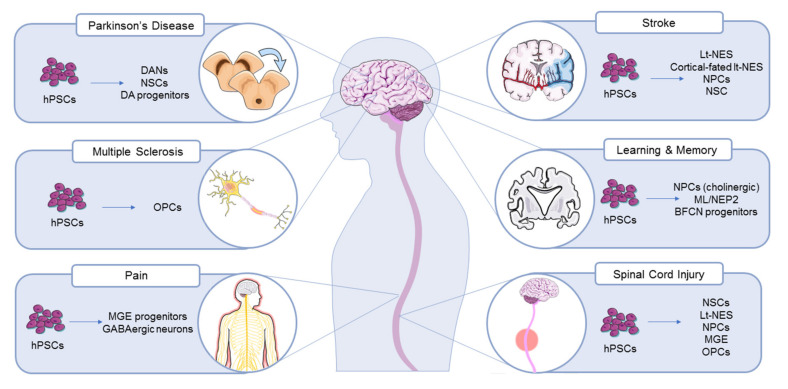
HPSC-based therapies for the treatment of neurodegenerative diseases. An overview of the different cell types generated from hPSC currently being studied and developed as cell therapies for the treatment of various neurodegenerative diseases.

Whilst human fetal material has long been explored for Parkinson’s disease, human embryos are not a viable source of material for cell therapy. Various human stem cell derivatives, the most common being neural stem cells (NSCs) or neural progenitor cells (NPCs) and DANs, have already shown an improvement in symptoms following cell transplantation in rodent and primate PD models (Figure 1, Table 1).

A pioneering study used a co-culture system of telomerase-immortalized human fetal midbrain astrocytes with hESC to achieve high-efficiency dopaminergic neurogenesis in hESC. This highly enriched DANs population provided significant functional benefit when transplanted into the striatum of the 6-hydroxydopamine (6-OHDA)-lesioned rat model of PD [13]. While this study represented an important steppingstone in the development of a stem cell-based therapy for PD, it also raised the concern of tumor formation due to the presence of proliferating undifferentiated neuro-epithelial cells in the graft.

Prolonged maturation of hESC-DANs in vitro prior to transplantation was showed to reduce the risk of tumor formation without affecting their function and efficacy in a primate model of PD [14]. More efficient and defined protocols, based on dual SMAD inhibition and activation of the Wnt signaling, have also been developed. Midbrain DANs expressing tyrosine hydroxylase (TH, the rate limiting enzyme in the synthesis of DA) were derived from hESC. Their function and efficacy in vivo was demonstrated in PD models using three host species. HESC-midbrain DANs engrafted in 6-OHDA-lesioned mice and rats and their survival was associated with an amelioration of amphetamine induced rotation behavior and bradykinesia. Scalability of the procedure was confirmed in parkinsonian monkeys and safety was suggested by the lack of tumor formation in the three animal models [15]. A similar approach is based on dual SMAD inhibition, modulation of the Wnt signaling pathway and embryoid body (EB) formation to direct hESC differentiation toward a ventral mesencephalic (VM) fate and generate an enriched population of floor-plate progenitors. When transplanted into the brain of a rat model of PD, hESC-derived VM progenitors differentiated into functional midbrain DANs, released DA in vivo 18 weeks post-transplantation and reversed motor impairments (amphetamine-induced rotational asymmetry) in 6-OHDA-lesioned rats [16] with an efficacy comparable to that seen with human fetal VM cells [17]. HESC-DANs are capable of long-distance innervation of the host brain [17]. However, the overall proportion of hESC-derived DANs within the grafted region remains low, due in part to their poor survival immediately following transplantation. Glial cell line-derived neurotrophic factor (GDNF) has recently been shown to increase the survival, plasticity, and functional integration of hPSC-derived VM DA progenitor grafts, in a time post-transplantation-dependent manner [18]. Interestingly, a new cell type resembling perivascular-like cells, called vascular leptomeningeal cells (VLMC), has very recently been identified in hESC-DANergic grafts [52]. The contribution of this population to the graft survival and function remains to be determined. 

Autologous human induced pluripotent stem cells (hiPSCs) cells potentially represent a more tolerable cell transplantation option than hESC and have attracted significant interest [19,20,21,23]. Han et al., 2015 confirmed that hiPSC-derived NSCs integrated into the host brain circuitry and differentiated to DA neurons when transplanted into the striatum of 6-OHDA PD rats. Functional recovery was evident from the 4th to the 16th week after transplantation. However, a greater proportion of NSCs differentiated to astrocytes than neurons, with efficiencies of 51.1% and 20.5% respectively [22]. Other studies avoided this issue by directly transplanting hiPSC-derived DANs into the striatum of 6-OHDA rats, observing functional recovery using the amphetamine-induced rotation test and highlighting no difference in results when comparing DANs derived from healthy or PD hiPSC lines [19,20]. PD-hiPSC-DANs do not show increased vulnerability to external toxic stresses compared to those generated from healthy individuals. Furthermore, when transplanted in the brain of α-synuclein overexpressing transgenic mouse model of PD [53], PD hiPSC-derived DANs did not exacerbate pathological α-synuclein accumulation in the host brain or in the grafts, suggesting that hiPSC derived from PD patients are suitable for autologous cell transplantation [20]. A reduction in motor deficits was also observed after transplantation of NPCs or DANs from hiPSC into the right side of the rat striatum [21]. However, three of twelve grafted rats showed tumor growth and subsequent death before eight weeks suggesting the presence of oncogenic factors or remaining undifferentiated cells. Despite the differences in the nature of the cell transplanted, these studies in rodents demonstrated functional recovery. Therefore, transplantation of hPSC-derived neuronal cells represents a potential therapeutic strategy to treat PD. 

In addition to rodent models, non-human primate (NHP) models of PD have been used to test hiPSC-based therapy. Following bilateral transplantation of hiPSC-derived NPCs into the putamen of MPTP (1-methyl-4-phenyl-1,2,3,6-tetrahydropyridine)-treated cynomolgus monkey, cells survived for six months and differentiated into DANs. A single parkinsonian monkey was transplanted and behaviorally assessed for skilled reaching. Results showed a slight functional improvement [23]. A subsequent analysis from the same group found improved neurological rating scores and higher recovery rates after transplantation of hiPSC-derived DA progenitors into the putamen of MPTP-treated cynomolgus monkeys. HiPSC-DA progenitor cells matured and survived for at least 12 months after transplantation and the efficacy of the cell therapy was similar to that of L-DOPA treatment. Moreover, the effects were similar whether the donor cells were from healthy individuals or PD patients [24]. Following this study in NHPs, which was a simulation of a planned clinical trial and confirmed the efficacy and safety of hiPSC-derived DA progenitors, a Phase I clinical trial in human PD patients was initiated in 2018 in Japan, and others are planned for the near future in the US and in Europe [7,54,55]. Current clinical trials using hPSC-derived neuronal cells for neurodegenerative diseases/injuries are summarized in Table 2.

Together, these studies build on evidence that an hPSC-based therapy has considerable potential for reversing motor defects of PD patients. The replacement of DANs from the substantia nigra by hPSC-DANs restores the deficit of dopamine release, subsequently stimulating the medium spiny neurons of the striatum and alleviating PD disabilities that include bradykinesia, resting tremor and muscle rigidity. Cell replacement therapy also could reduce the side effects of medication such as dyskinesia (uncontrolled involuntary movements) resulting from the long-term use of Levodopa. A major limitation of such treatment however is its sole focus on motor symptoms, which comprise only part of PD pathology. It remains unclear whether any of these treatments will have an impact on the non-motor symptoms of PD including mood disorders, such as depression, and cognitive decline.

### 2.2. Stroke

**Figure 2:**
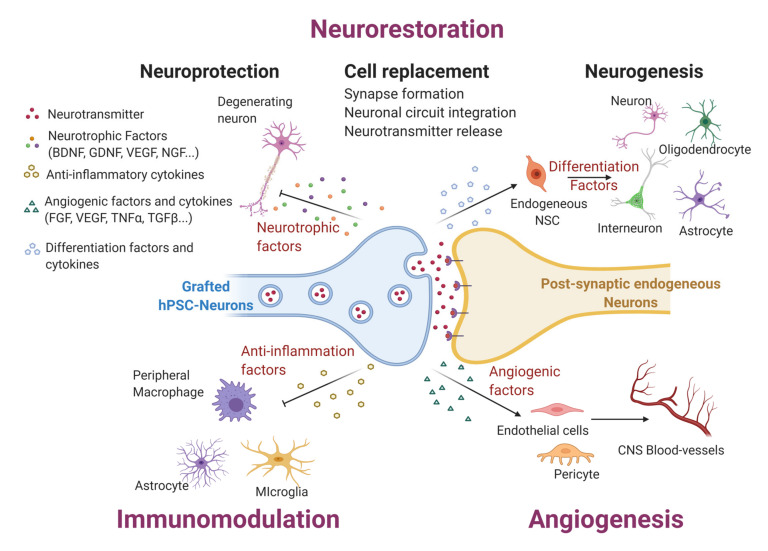
Potential mechanisms of hPSC-based therapies for neural tissue regeneration.

Despite stroke being a significant cause of disability in adults [56], outside of long-term rehabilitation, few therapeutic strategies exist to treat the condition. Stroke is characterized by a sudden interruption of blood flow in a particular area of the brain. It can lead to death of a range of neuronal cell types with limited regeneration potential and therefore, the use of hPSCs as a cell-based replacement therapy is a promising potential approach to promote recovery. Transplantation of neuronal cells derived from hPSCs can lead to functional improvement in animal models of stroke through several mechanisms including neuronal replacement, modulation of inflammation, neuroprotection and stimulation of angiogenesis [57] (Figure 2).

Due to the heterogeneity of cells lost in stroke, several different cell types have been used for transplantation therapies (Table 1, Figure 1). It remains unclear which of these cell types is most advantageous.

Long-term self-renewing neuro-epithelial-like stem (lt-NES) cells are multipotent progenitors that can give rise to glia and neurons and are most similar to the early neuroepithelium of the developing embryo. Lt-NES cells can be derived from human iPSC or ESC [58]. In contrast to differentiated cells that are already specialized, lt-NES cells are less lineage-restricted and can be instructed by local factors in vivo to further differentiate into the required neuronal lineages. This could have a major advantage in stroke where several neuronal cell types are damaged and directed differentiation to replace only one type of neurons might not provide maximum benefit. Previously, hiPSC-derived It-NES have shown some therapeutic promise following transplantation into the striatum or cortex of mice and rats that with stroke-damage from middle cerebral artery occlusion (MCAo) [25,26,27]. Recovery of movement was observed as shortly as one week after transplantation, suggesting that this rescue may be due to neurotrophic factors release, such as vascular endothelial growth factor (VEGF), rather than neuronal cell replacement. Transplanted lt-NES cells differentiate into several neuronal subtypes including cortical neurons, and demonstrate functional characteristics of neurons, such as action potential generation with integration into the host circuitry evident five months post-transplantation. Motor deficits were consequently attenuated [25]. HiPSC- lt-NES which had been fated towards a cortical phenotype in vitro prior to transplantation showed less proliferation and more efficient conversion to mature neurons than non-fated cells [27]. Six months post-injection, transplanted hiPSC-fated cortical neurons receive direct and functional synaptic inputs from the stroke-injured brain [26], their axons are myelinated in the host brain and their terminals form excitatory glutamatergic synapses with host cortical neurons [28]. Furthermore, physiological sensory stimuli can modulate their spontaneous activity [26] and their activity can maintain normal motor function in MCAo rats [28], demonstrating functional integration of the grafted hiPSC-cortical neurons to the stroke-injured brains’ neural circuitry. Whilst these studies show that lt-NES cells generally become committed and lose proliferative capacity in vivo, some cells remain proliferative and potentially tumorigenic. As such, the risk of graft overgrowth is a substantial concern.

As an alternative strategy, hiPSC-derived NPCs have been transplanted into stroke-damaged rodents and shown to be equally as effective in promoting functional recovery. Neuro-progenitor cells differ from IT-NES by their more limited capacity to proliferate, although they retain multipotency. This characteristic may help to mitigate the problem of graft overgrowth observed with It-NES and a number of studies have claimed significant success with this strategy in both mice and rats. NPCs (of telencephalic fate) transplanted into the striatum of ischemic mice survive, migrate and are associated with an improvement of modified neurological severity score (mNSS) for up to six weeks post-transplantation. The functional recovery observed in the transplanted group could be explained by neural circuit reconstitution [29]. Similar results were found using the adhesive removal test where the latency/speed of removal is measured. HiPSC-NPCs transplantation not only enhanced sensorimotor functional recovery but also reportedly increased trophic support and improved blood flow in the cortex by restoring neurovascular coupling up to 28 days post-transplantation. However, the study did not assess the relative contributions of different neural cell types to recovery [30]. Similarly, hiPSC-NPCs transplantation into the brain of stroke damaged rats also leads to functional improvement in both rotarod and stepping tests, with initial motor recovery from one-week post-transplantation. A more gradual recovery was however observed in two other metrics, the apomorphine-induced rotation tests and the mNSS. The study highlighted the short-term effects of hiPSC-NPCs and demonstrated their different mode of actions by showing the formation of neuronal tissues from the transplanted hiPSC-NPCs and the reduction of inflammation, gliosis and apoptosis in the damaged brain [31]. More recently, improvement in limb asymmetry was achieved by transplantation of hiPSC-NPCs into the right cortex of MCAO stroke-damaged rats. However, there were only modest improvements in motor function, suggesting further clinical development would be required [32]. 

To date, only one study involving the use of hiPSC has been carried out in a non-rodent stroke model. HiPSC-NSCs were transplanted into the brain parenchyma surrounding MRI-identified lesions of an ischemic pig stroke model and were shown to survive long-term, differentiate to neurons and oligodendrocytes and improve tissue recovery in the damaged brain. Metabolic effects were recorded post-transplantation, such as reduced changes in white matter integrity, enhanced cerebral blood perfusion and recovery of brain metabolism in the damaged tissue. HiPSC-NSC post-stroke transplantation also lead to neuronal protection and reduced microglial activation. However, no confirmation of functional recovery was assessed in this study [33].

A limitation of the field is that only short-term studies have been carried out to date. Longer-term functional follow-ups are necessary to provide evidence of therapeutic efficacy. Stroke is also a difficult disease to model as the pathology depends on the brain regions affected. From these studies it remains unclear whether functional recovery could be attained in all brain regions and the extent to which the degree of neuropathology alters the results. 

### 2.3. Epilepsy

Epilepsy is a common neurological disorder which manifests by an excessive, rhythmic (ictal) and abnormal brain activity causing spontaneous recurrent seizures, unusual behavior or sensations and loss of consciousness. Dysfunction of GABAergic inhibitory interneurons plays a major role in the pathological hyper-excitability underlying epilepsy, owing to their responsibility for dampening neuronal activity and promoting network inhibition. Inflammation also has a significant role in epilepsy. However, there is an active debate as to whether inflammation is the cause or consequence of epilepsy [59,60]. Although many anticonvulsants drugs are used in the treatment of epilepsy, their efficacy varies greatly between individuals and adverse effects are near ubiquitous amongst patients [61]. Moreover, many patients cannot be successfully stabilized on anticonvulsants, necessitating drastic surgery such as surgical resection of the epileptic focus (the center of epileptic rictal activity). Consequently, the development of a stem cell-based therapy could represent a great advantage for the treatment of epilepsy. 

HESCs can be differentiated into medial ganglionic eminence (MGE) cells, precursors of GABAergic interneurons (GINs). Significant cognitive improvement was observed after transplantation of hESC-MGE progenitors into the hippocampus of a pilocarpine-induced temporal lobe epilepsy (TLE) mouse model. Grafted hESC-MGE progenitor cells differentiated into active GABAergic neurons with mature firing patterns, showing their integration into the host hippocampal circuitry and suggesting a synaptic mode of action [34,35]. Alternatively, hESC-interneuron grafts can ameliorate memory impairment through release of GABA or neurotrophic factors that protect hippocampal neurons and synaptic integrity, or by releasing factors that reduce inflammation which is known to impair learning and memory [62,63].

In one of the studies, transplanted cells were also functionally effective at reducing seizure activity for up to four months post-transplantation, which was directly attributed to transplant-derived inhibitory currents passed onto endogenous hippocampal neurons [34]. However, the more recent study did not show significant seizure suppression after transplantation of hESC-GABA neurons progenitors into the brain of TLE mice compared to media injection [35]. Moreover, in this study, tumor-like NSC clusters were also observed in some injected mice, likely due to proliferative undifferentiated cells remaining in the transplanted population. The discrepancies between the two studies might therefore be explained by the difference in heterogeneity of the transplanted cell populations. Although hPSC-based therapies have great potential in the treatment of epilepsy, further studies are necessary in both rodent and non-rodent models to prove their efficacy in suppressing seizures and importantly to ascertain the safety of the procedure.

### 2.4. Learning & Memory Disorders/Dementia (Alzheimer’s Disease)

Learning and memory are complex cognitive processes involving hippocampal function that are severely affected by aging and some neurodegenerative disorders such as Alzheimer’s disease (AD). AD is the most common age-related form of dementia and is clinically characterized by a progressive loss of cholinergic neurons and synapses, deposition of neurotoxic proteins such as extracellular amyloid-β (Aβ) plaques and intracellular neurofibrillary tangles (NFTs) [64]. While AD is currently untreatable, increasing evidence supports the therapeutic potential of regenerative medicine to treat AD.

Over the last decade, numerous preclinical stem cell therapies have been attempted to replace lost neurons or support existing neurons in animal models of AD. NSCs derived from mouse adult brain or ESC were transplanted into AD rodent models and resulted in generation of cholinergic neurons, increased synaptic strength and enhancement of memory performance [65,66,67]. Improvements in learning and memory abilities have also been demonstrated after hPSC-based cell replacement therapies (Table 1, Figure 1). HiPSC-NPCs of cholinergic phenotype were transplanted into the hippocampus of the PDAPP (PDGF promoter driven amyloid precursor protein) transgenic mouse model of dementia [68]. By 45 days post-transplantation, hiPSC-NPCs were found to survive and differentiate into cholinergic and GABAergic neurons in the host brain, resulting in an improvement in spatial memory [36]. Mouse and human ESC were also directed to differentiate into basic forebrain cholinergic neurons (BFCN) progenitors and transplanted into the forebrain of AD mouse models. Two months after the injection, transplanted BFCN progenitors predominantly differentiated into mature cholinergic neurons that functionally integrated into the host endogenous cholinergic circuitry. HESC-BFCN therapy could restore cholinergic function and alleviate cognitive deficits of two strains of AD mouse models (5XFAD and APP/PS1) up to six months post-transplantation [37]. These results demonstrate great potential for hPSC transplantation to improve learning and memory disorders such as AD. 

Accumulating evidence suggests that stem cells transplantation influences the pathological features of AD through multiple modes of action. Similar to stroke, the therapeutic potential can be partly attributed to a paracrine effect of neurotrophic factors (in this case BDNF and GDNF) that ameliorate various cellular functions in animal models of AD, including neural integrity, endogenous neurogenesis, microglial activity, angiogenesis, mitochondrial function, autophagy and apoptosis [69,70] together improving cognitive function. Of interest, while Huntington’s disease (HD) is commonly referred as a motor disorder, cognitive impairments are present and often progress to dementia. Similar behavioral improvements have been found after transplantation of mouse and human iPSC-NPCs into the striatum of *YAC128* HD mice [71,72]. Transplanted cells were shown to differentiate into medium spiny neurons [71], the most affected neuronal cell type in HD, as well as GABAergic neurons [72]. HPSC-NPC may also represent an effective neuronal cell replacement therapy for HD. 

While most NPC/NSC and BFCN transplantations were successful at improving cognitive dysfunction in AD animal models, they failed to reduce the level of Aβ plaques in the AD brain. Following a different strategy, hiPSC-derived macrophage-like (ML) cells were generated and engineered to express *Neprilysin-2* (*NEP2*), a secretable protease with Aβ-degrading activity. HiPSC-ML/NEP2 were injected into the hippocampus of the 5XFAD transgenic AD mice [38,73]. Although the effect on cognitive function and neuronal damage was not examined, a significant reduction in the level of Aβ was observed in the transplanted mouse brain. The reduction of Aβ was not significant when unmodified hiPSC-ML were transplanted, demonstrating that the secretion of NEP2, and not phagocytosis by hiPSC-ML, caused the elimination of Aβ. While further investigations are necessary to evaluate their protective effect on neuronal damage and subsequent cognitive decline, this study suggests a potential therapeutic benefit of NEP2-secreting hiPSC-ML for the treatment of AD. 

### 2.5. Multiple Sclerosis

Multiple Sclerosis (MS) is a chronic demyelinating disease that affects the CNS. Current therapies are primarily directed against the immune system to treat the inflammatory component of the disorder. However, the real challenge is to develop re-myelinating and neuro-protective therapies. 

To establish a potential source of myelinogenic oligodendrocytes for the treatment of MS, several protocols were developed to generate oligodendrocyte precursors cells (OPCs) from hiPSC [39,74,75]. HiPSC-OPCs were transplanted into the corpus callosum of a genetic model of congenital hypomyelination, the shiverer mouse [76]. In vivo, hiPSC-OPCs differentiated into oligodendrocytes that produced myelin and had the ability to efficiently myelinate the hypomyelinated shiverer brain with no evidence of tumorigenesis at nine months post-transplantation. Furthermore, the transplants led to a significant functional improvement and significantly increased lifespan by ~20% compared to their untreated counterparts [39]. Importantly, when hiPSC were derived from four progressive MS patients they could also be induced to form highly enriched populations of OPCs. Although no functional assay was performed in this study, immunohistochemistry demonstrated that the transplanted hiPSC-OPCs differentiated, expressed mature oligodendrocyte markers after transplantation in vivo and myelinated axons in an immuno-compromised shiverer mouse brain, highlighting the potential of autologous hiPSCs to treat MS [40].

### 2.6. Spinal Cord Injury

Spinal cord injury (SCI) often results in catastrophic neurological deficits which dramatically diminish the quality of life of affected individuals. Surgery (laminotomy which involves the surgical removal of bone to relieve pressure in the spinal canal) and rehabilitation are the only interventions commonly used to improve functional recovery after SCI. The corticosteroid methylprednisolone is the only pharmacotherapy currently approved and has been used to reduce inflammation in the spinal cord (SC) acutely after injury. However, it has limited efficacy and serious side-effects, such as gastrointestinal hemorrhage and respiratory bacterial infection, and this medication is no longer routinely provided [77]. Despite decades of efforts to develop effective treatments, there is still an urgent need for new therapeutics that promote functional recovery after SCI. 

Central to the pathology of SCI is a major insult to the nervous system. A trauma of the spinal cord can cause acute damage to ascending and descending tracts and lead to axotomy (severing of axons). Immediately after primary injury, a robust neuro-inflammatory response occurs and triggers the secondary injury mechanisms in the chronic phases of SCI, leading to cell death and further tissue degeneration (Wallerian degeneration). Around the site of the injury, the formation of a cyst and a growth inhibitory scar (known as the glial scar) will prevent tissue regeneration [78]. For these reasons, stem cells, in particular hiPSC, have attracted great interest as a potential source for cell replacement therapy after SCI. 

Human iPSC-derived neurospheres were transplanted into a mouse model of contusive SCI at the T10 level [41]. The cells differentiated to neurons, oligodendrocytes and astrocytes. Functional recovery was evident 112 days after injury in multiple motor parameters including gait and overall locomotion. However, only 22% of the cells differentiated into mature neurons. In a further study using hiPSC-lt-NES transplanted in a mouse at the T9 level, 75% of transplanted cells differentiated to neurons. Functional recovery of the hindlimb was found using the Basso Mouse Scale (BMS) and was associated with improvements in the motor evoked potential (recorded from the hindlimb and stimulated in motor cortex) [42]. 

HiPSC-NPCs have also been transplanted into an immuno-competent mouse model of SCI but showed very limited survival, no reduction of the size of the lesion and no functional recovery [43]. In contrast, functional improvement of hindlimb dysfunction and structural recovery of the spinal cord were evident following transplantation of hiPSC-NPC in a mouse study [44]. The same discrepancies were reported in rat models of SCI. HiPS-NPCs were injected into C5 lesion sites of immunodeficient rats. Although the majority of cells differentiated to neurons whose axons were found to have integrated and formed synapses with host neurons three months post-transplantation, there was no functional improvement (assessed by the grid-walking, grooming, and vertical exploration) [45]. In contrast, a study with a much larger sample size (34 transplanted and 35 control rats) showed clear functional motor improvement (measured by the Basso, Beattie and Bresnahan (BBB) locomotor scale method, beam walking, rotarod and plantar test) after transplantation of hiPSC-NPCs at the T8 level of the spinal cord. After 17 weeks, hiPSC-NPCs differentiated to astrocytes, oligodendrocytes and specific neurons (interneurons, dopaminergic, serotoninergic and motor neurons), but a large portion of the grafts was glial cells. Interestingly, human ChAT (cholineacetyltrabsferase) positive motor neurons were found in the ventral part of the spinal cord while human calbindin expressing interneurons were localized in the central part of the SC, showing that the cells can migrate to specific regions of the tissue and adopt specific phenotypes [46]. Despite promising results, the degree of functional recovery after stem cell transplantation remains modest. Recently, *Notch* activation induced by injury in the SC has been shown to orient transplanted hiPSC-NPCs towards astrocyte lineage and reduce their therapeutic efficiency [79]. Remarkably, modulation of notch signaling by GDNF in transplanted cells increased their neuronal fate and enhanced their electrical integration independently of an effect on cell survival. This strategy resulted in an improved functional recovery after transplant and represents an important optimization of hiPSC-NPCs therapy for SCI. 

HiPSC-NSCs have also been trialed as cell therapy in a marmoset model of SCI. Injury was induced at the C5 level of the spinal cord and behavioral analyses were performed for up to 12 weeks afterwards. Functional recovery was observed in motor parameters such as open field, bar grip and cage climbing tests. However, although transplanted cells were found to differentiate into all three lineages (neurons, astrocytes and oligodendrocytes), approximately one quarter of the cells remained immature. Despite this limitation, no tumorigenicity was observed in the limited time frame of the study [47]. Longer and additional studies in large animals would be required to reinforce the current evidence.

Because re-myelination of axons is an essential component of the recovery, others have evaluated the therapeutic potential of OPCs, derived from hESC or hiPSC, for the restoration of neuronal pathways after moderate contusive SCI in rats. In both cases, most cells differentiated to mature oligodendrocytes expressing Myelin Basic Protein (MBP) and integrated in the host spinal cord. Transplanted 2 h after injury, hESC-OPCs lead to an improvement of somatosensory evoked potential (SSEPs) recorded at the cortex showing functional improvement of sensory pathways [48]. Transplantation of hiPSC-OPCs 24 h after injury resulted in a reduction of the cavity size and glial scarring at the injury site. A significant increase in number of myelinated axons was also reported. Although the mechanisms involved are still unclear, hiPSC-OPCs improve recovery of motor function (measured using the BBB scale) after transplantation into SCI [49]. Of note, mouse iPSC- NSCs derived from both wildtype and shiverer mice were transplanted into the spinal cord of a mouse model of SCI at the T6 level. While both cell lines integrated and differentiated into oligodendrocytes, astrocytes and neurons, wildtype-derived cells demonstrated a much greater improvement in locomotor function, demonstrating the key role of re-myelination in functional recovery of the spinal cord [80]. 

Lastly, some investigations have focused on other pathological aspects of SCI, which include neurogenic bladder disorders and neuropathic pain. A shared hallmark of both conditions is the loss of GABAergic inhibitory tone in the injured spinal cord [81,82]. HESC were induced to form MGE progenitor cells and transplanted in the lumbar enlargement of SCI mice. By six months post-transplantation, hESC-MGE progenitors integrated and differentiated into mature GABAergic neurons and glial cells. HESC-MGE grafts improved neurogenic bladder dysfunction and relieved central neuropathic pain, two of the most debilitating SCI-related symptoms [50]. 

Despite all preclinical studies performed in rodents to establish an hPSC-based approach for spinal regenerative medicine, clinical trials using hPSC to target SCI have not been fully conducted. The Food and Drug Administration (FDA) approved the first clinical trial in the US for the use of hESC-derived oligodendrocytes to treat SCI. Geron Corporation started the trial on 4 patients in 2010 but it was later closed for business reasons. Asterias Biotherapeutics re-initiated a Phase 1/2a Dose Escalation Study with 25 patients in 2015 but, to date, no results have been reported (Clinical Trial ID NCT02302157, Table 2). Clinical trials using transplantation of hiPSC-NPCs are also scheduled to start in Japan [8].

### 2.7. Neuropathic Pain

Chronic neuropathic pain is an exacerbated or prolonged pain caused by damage or disease affecting the somatosensory nervous system (including nerve injury, cancer, diabetes or viral infection). Chronic neuropathic pain can be classified as a neurodegenerative disease [83,84] that culminates in decreased central inhibition in the spinal cord [85,86,87]. 

Neuropathic pain is currently treated with non-specific management strategies such as anti-epileptics, antidepressants and, in some cases, opioids. However, these treatments are known to have poor efficiency, produce undesirable side effects and/or long term addiction [88,89]. Cell replacement therapies to treat neuropathic pain have been explored as a means to increase GABA locally at the site of central dysfunction. Functional replacement of spinal GABAergic inhibitory neurons has been initially performed by the transplant of mouse embryonic MGE progenitor cells and resulted in attenuation of neuropathic pain by restoring central inhibition through release of GABA [90,91]. HESC-MGE progenitor cells have also been transplanted into the mouse spinal cord and shown to alleviate central neuropathic pain following SCI [50]. Most recently, we have generated matured functional GABAergic neurons from hiPSC and transplanted them into the spinal cord of mice with established peripheral nerve injury, providing pain relief for up to two months without damaging the spinal cord or affecting the mice motor function [51]. Given the potent analgesic effect of the GABAergic transplants and the resultant potential for its clinical use, a long-term study is currently underway to ascertain the efficacy and safety of this procedure. However, a limitation of this strategy is the control of GABAergic neurons migration and GABA release. An excessive restoration of central inhibition in the spinal cord, especially in the ventral horn, could potentially affect motor function and lead to debilitating disorders.

## 3. Current Challenges 

**Figure 3:**
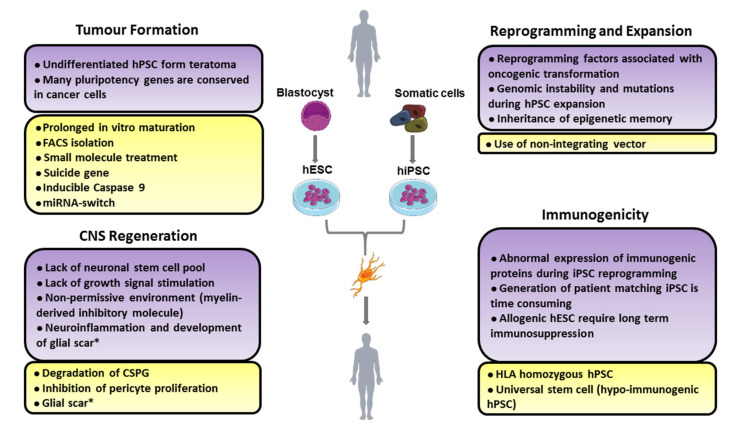
Challenges in clinical application of hPSC transplantation for the treatment of neurodegenerative diseases. In purple are summarized the current issues associated with the use of hPSC in regenerative medicine of the CNS, in yellow the potential solutions. * Note contradictory findings regarding the role of glial scar in CNS regeneration.

The challenges in the clinical application of hPSC transplantation for the treatment of neurodegenerative diseases or conditions fall broadly into two categories: issues with hPSC transplantation generally (including hPSC generation and differentiation), and those specific to the regeneration of the central nervous system (Figure 3).

### 3.1. Transplantation of Stem Cells

#### 3.1.1. Pluripotency and Cancer

Pluripotent stem cells have the unlimited capability to self-renew and differentiate into virtually all cell types of the body. These attributes make them an attractive candidate for cell replacement therapies and hPSC hold much hope for regenerative medicine. However, when transplanted in vivo undifferentiated hPSC form teratomas and the risk of tumor formation has largely restricted the clinical application of hPSC. 

Pluripotent stem cells share a number of characteristics with cancer cells. Both fundamentally possess an indefinite capacity to proliferate, the ability to bypass DNA repair checkpoints [92,93] and express oncogenic markers. For example, c-MYC transcription factor (TF) is highly expressed in both cancerous cells [94,95,96] and hESC [97,98] and is central for generating iPSC [99,100]. Recently, *c*-MYC has been identified as the major oncogenic effector of Wnt/β-catenin signaling in hESC tumorigenicity [101]. Many of the genes and networks associated with pluripotency are also conserved in cancerous cells. The key pluripotency marker *OCT4* is involved in the development of multiple cancers, such as ovarian [102,103], cervical [104], colorectal [105,106], liver [107], and oral cancer [108]. Generally, *OCT4* expression correlates with worse cancer outcome in most tumors [109], while its down-regulation is associated with slowed tumor progression [110]. *OCT4* is not the only important pluripotency factor. It forms a complex with other TFs such as *SOX2* and *NANOG* to regulate the expression of different genes and maintain ESCs in an undifferentiated state. *NANOG* also plays a role in the self-renewal of CD24+ cancer stem cells in hepatocellular carcinomas [111] and has been found aberrantly expressed in a variety of human cancers, including head and neck squamous cell carcinomas (HNSCC) [112,113]. *SOX2* has been shown to be expressed in at least 25 different types of cancer and to drive cancer cell survival [114]. Another stemness factor, *KLF-4* (*Krüppel-like factor 4*), has been reported to promote DNA repair checkpoint uncoupling and cellular proliferation in breast cancer by p53 suppression [115]. *KLF4* also acts as an oncogene in Glioblastoma by supporting glioblastoma stem cells survival and promoting their proliferation [116]. Pluripotent stem cells are also known to possess many of the hallmarks of cancer, including lack of contact inhibition in vitro [117], loss of *p53* [118,119] and *Rb* (retinoblastoma tumor suppressor protein) inactivation regulating their cell cycle [120]. Furthermore, long-term culture generates cytogenetic abnormalities and stem cells are highly susceptible to acquire mutations that are advantageous to regrowth. Given the high genomic instability of hPSC, oncogenic mutations are likely to accumulate over time during the production of large quantities of cells needed for cell therapy. Notably, 44% of genes up-regulated as a result of genomic instability in hESCs were functionally linked to genes commonly expressed in a range of cancers [121]. An important example is the spontaneous acquisition of *p53* mutations by both hiPSCs and hESCs in prolonged culture, similar to the ones observed in human cancer [9]. This may result in a high proportion of potentially tumorigenic cells within the culture, making the cells unsuitable for transplantation in humans. 

After hPSC differentiation, residual undifferentiated or partially differentiated cells may remain and induce tumor formation when implanted into animal models. In the case of neurodegenerative diseases, the risk is not only the development of teratomas from undifferentiated hiPSC/hESC but also undifferentiated neural tumors, whether primitive neuroectodermal tumors or gliomas, from partially differentiated neuronal cells. Persistent proliferation and tumorigenic masses were observed after transplantation of hPSC neuronal derivatives in rodent models of PD [13,21], epilepsy [35], and SCI [122], which highlights the importance of developing efficient, safe and clinically relevant protocols for complete elimination of undifferentiated cells.

#### 3.1.2. Methods to Prevent Tumor Formation

In an effort to overcome this issue, several strategies have been explored for the development of safer stem cell therapies. Prolonged maturation of hPSC-DANs in vitro prior to implantation has been shown to reduce graft overgrowth and tumor formation in primate model of PD brain [14]. However, although this study showed behavioral improvements of monkeys transplanted with mature hESC-DANs, the elimination of undifferentiated hESC could not fully prevent the risk of tumor formation due to persistent proliferating immature neural cells. Others have implemented a purification step by fluorescence activated cell sorting (FACS) to select more mature neuronal cells for transplantation, eliminate undifferentiated cells and avoid tumor formation [15,34,50,123]. Undifferentiated cells can also be eliminated using small molecules. Treatment of a hESC-derived population with chemical inhibitors of Survivin signaling (quercetin or YM155) induced selective apoptotic cell death of undifferentiated hPSCs and was sufficient to prevent teratoma formation after hPSC transplantation [124]. Inhibition of β-catenin signaling with chemical inhibitor FH535 in hESC reduced teratoma formation by 79%. Although the β-catenin pathway plays a fundamental role in hESC self-renewal and maintenance of stem cell properties, its inhibition in hESC before induction of differentiation did not compromise their pluripotency [101]. Similarly, pre-treatment of hPSC-NPCs with γ-secretase inhibitor, an inhibitor of Notch signaling, decreased the proliferative capacities and promoted differentiation of undifferentiated neural cells, thereby preventing tumor formation upon transplantation into SCI model rodents [125].

Suicide genes have also been employed for the elimination of undifferentiated and potentially tumorigenic cells. Herpes simplex virus thymidine kinase (HSV-TK) is the most commonly used suicide gene in hESC/hiPSC and has proven efficient in eliminating tumor formation after transplantation [126,127,128,129,130]. However, of HSV-TK substrate ganciclovir has a poor capacity to cross the blood-brain barrier (BBB) [131] and its long-term administration is associated with potential health risks associated with, such as impairment of renal function, hepatic dysfunction, and pancytopenia [128]. The use of HSV-TK may therefore not be suitable in cell therapy, especially for neuronal disorders. Moreover, by targeting DNA synthesis, HSV-TK has the ability to induce apoptosis specifically in highly proliferative cells but may not be efficient to eliminate slowly dividing cells. A cell-cycle-independent fail-safe strategy using an inducible caspase 9 (iC9) gene has also shown its efficacy in ablating teratomas derived from iPSCs [132,133,134] or tumors originating from malignant transformation of transplanted hiPSC-NPCs in a mouse model of SCI [135]. A specific chemical inducer of dimerization (CID, AP1903) activates iC9 which subsequently triggers an apoptotic response that kills hPSCs or their derivatives within 24 h. A less conventional method of “microRNA switch” (miR-switch) has also demonstrated success. MicroRNA-302-5p (miR-302a) is highly expressed in undifferentiated hPSCs but progressively decreases upon differentiation to become undetectable in fully differentiated cells. A miR-switch based on miR-302a activity was developed to identify residual undifferentiated and partially differentiated cells following differentiation of hiPSCs. Selective hiPSC elimination was achieved by controlling puromycin resistance using the miR-302a switch and prevented teratoma formation in an in vivo tumorigenicity assay [136].

All these approaches provide a safeguard for clinical use of hPSC-cell therapies. However, because most of hPSC transplantation studies are performed in immune-deficient animals or in presence of immune-suppressive drugs, the real risk of tumor formation in immune-competent hosts is mostly unknown. Recently, using multiple models of humanized mice it has been suggested that autologous hPSC-derived therapies are unlikely to form teratomas in the presence of natural killer (NK) cells [137]. 

#### 3.1.3. Oncogenic Risks Associated with Reprogramming

Despite their obvious advantage, using hiPSC in regenerative medicine implies additional risks. Genome-wide studies have unveiled numbers of large and point mutations in hiPSC, leading to serious doubts regarding their safety and potential clinical applications [138,139]. It is therefore critical to evaluate the safety of hiPSC (and their derivatives) generated prior to transplantation. Following this idea, oncogenic mutations were detected in the hiPSCs reprogrammed from the somatic cells of the second patient enrolled in the clinical trial testing hiPSC-derived RPE as cell replacement therapy for macular degeneration. As a result, the transplantation of the second patient was postponed and the trial terminated. The mutations were not detectable in the patient’s original fibroblasts [4]. 

Reprogramming adult somatic cells to a state of pluripotency requires their exposure to multiple reprograming factors (Oct4, Sox2, Klf4, c-Myc or Nanog and Lin-28) which are critical for both the acquisition and maintenance of induced pluripotency but can promote oncogenic transformation as previously discussed. The process of reprogramming itself also induces genomic alterations. Single nucleotide variations (SNVs) sometimes occur in the coding region of a gene, resulting in the expression of a mutated protein that can lose or gain function and become potentially harmful. Copy-number variations (CNVs), involving the duplication or deletion of a large portion of DNA, can also be generated by reprogramming, causing potentially dangerous mutations in different genes [140]. Furthermore, the first generations of iPSCs were reprogrammed using retrovirus as delivery method for reprogramming factors [100,141] which integrate into the genome of the cells and therefore can cause potentially dangerous mutations. Non-integrative reprogramming methods that reduce the occurrence of genetic variations [142,143,144] have since been developed and are a prerequisite for clinical use of hPSC. These include DNA-based methods such as Sendai virus vectors [145], episomal vetors [146] and excisable piggyBac vectors [147]. DNA-free reprogramming methods, including messenger RNA [148] and proteins [149,150], have also proven efficient and are often preferred as they are transitory methods and are eliminated rapidly. In contrast, NPC and DANs derived from hiPSC generated using lentiviruses exhibit residual expression of exogenous reprogramming genes. Furthermore, a protein-based reprogramming method leads to more robust hiPSC that are highly expandable without senescence [21]. Vector- and transgene-free hiPSC-NPCs were transplanted into a model of stroke [30] or PD [21,24]. No tumor formation was observed for up to 12 months post-transplantation. 

#### 3.1.4. The Epigenetic Landscape of Induced Pluripotent Stem Cells

To acquire pluripotency, a somatic cell needs to be reprogrammed from a differentiated state into a permissive state similar to the early embryo. During this process of reprogramming, the genome may permanently be altered through aberrant imprinting, activation of endogenous retroviruses [151], and incomplete reprogramming.

IPSC derived by reprogramming show DNA methylation signatures similar to their somatic tissue of origin, which promote their differentiation into lineages related to the donor cell and restrict their participation to other cell fates [152,153,154]. Reprogramming also generate aberrant differential methylation (often a reduction in DNA methylation) differing from the somatic donor cell or hESC [153]. In general, hiPSC retain their specific epigenetic signature across differentiation and as a result, were reported to have impaired differentiation potential compared to hESC [152,153,155]. While prolonged expansion of iPSC in culture can reset iPSC and allows the loss of this epigenetic memory [156], extended culturing also promotes the accumulation of genetic defects as discussed previously, and induces other epigenetic abnormalities such as chromosome X erosion in female hPSC [157,158]. In addition to causing differentiation defects, epigenetic alterations can confer a proliferative advantage to the cells, due for example to an increased expression of oncogenes located on the reactivated chromosome X [159], and potentially pose additional risks of cancer for the clinical application of hiPSC.

#### 3.1.5. Immune Rejection

A crucial step toward successful clinical application of hPSC is to overcome the immune response that may be evoked by their transplantation. Since their groundbreaking discovery by Shinya Yamanaka in 2006, iPSC have revolutionized regenerative medicine. In addition to avoiding the ethical concerns linked to the destruction of human embryos, the main advantage of hiPSC over hESC is that they can be derived directly from the patients themselves and be used for autologous cell replacement therapy. Unlike an allogenic graft using hESC, autologous transplantation with hiPSC would, in theory, elicit negligible immune response, eliminating the risk of graft rejection.

IPSC immunogenicity was not initially questioned as it seemed obvious that the reprogrammed iPSC carried the same markers of immunogenicity that they expressed in their original somatic state. Zhao et al., 2011 were the first to investigate the matter by testing the immunological reactions triggered by auto-graft of undifferentiated mouse and human iPSC in syngenic or humanized mice respectively [160,161]. Although autologous cells were generally assumed to be immune-tolerated by the recipient they originally came from, an unexpected immune response to teratomas formed from autologous murine or human iPSC (but not mESC) was reported and associated with an infiltration of T lymphocytes and necrosis [160]. Similarly, transplantation of autologous hiPSC-derived smooth muscle cells (SMCs) into skeletal muscles of humanized mice triggered an immunogenic response and resulted in T cells infiltration of the graft, while autologous hiPSC-derived retinal pigment epithelial (RPE) cells were well tolerated and showed low immunogenicity both in the eyes and skeletal muscles [161]. Because integration-free methods were used for reprogramming, immune-rejection could not be caused by malignant transformation due to vector integration. These results initially raised serious doubts about the possible use of hiPSC in regenerative medicine.

However, many studies have since counterbalanced this initial observation. Immunogenicity of endothelial, hepatocyte and neuronal cells derived from ESC and iPSC was tested by assessing cytotoxic T lymphocyte response in vitro and in vivo after transplantation in syngeneic mice. No immune response to syngeneic iPSC was observed [162]. Similarly, differentiated skin and bone marrow tissues derived from mouse iPSC showed no difference in the success rate of transplantation when compared to mouse ESC-derived tissues. Furthermore, no immune response, including T cell infiltration, was observed for tissues derived from syngeneic iPSC or ESC [163]. These results were further confirmed by the successful transplantation of porcine iPSC-NPC into the spinal cord of syngeneic mini-pigs in the absence of immunosuppression. Long-term survival with neuronal and glial differentiation and no immune rejection of the transplanted cells were noted [164]. More recently, patient-derived hiPSC-DAN progenitors were shown to trigger immune rejection after transplantation into allogenic humanized mice, but showed absence of immunogenicity when transplanted into patient-humanized (using the patient’s peripheral blood mononuclear cells) mice [7].

Several mechanisms such as epigenetic and genetic instability in hiPSC could explain immune responses in transplant recipients even when differentiated cells are transplanted. A host immune response (T-cell infiltration) to hiPSC appears to be dependent on the antigenic profile of the transplant. Misexpression of immunogenic antigens, Zg16 and Hormad1, was found in hiPSC-derived SMCs, but not in hiPSC-derived RPEs, and potentially explains the disparity in immunogenicity of these two hiPSC derivatives after transplantation in humanized mice [161]. First, epigenetic abnormalities observed in hiPSC could lead to abnormal expression of immunogenic proteins during specific lineage differentiation of hiPSCs but not hESCs. Second, genomic translocation or point mutations acquired during the process of reprogramming could create new immunogenic determinants. Importantly, despite the controversy, the lack of immunogenicity of hiPSC-derived cells has been confirmed in the patient who received his own hiPSC-derived RPE to treat macular degeneration [4] and in the Parkinson’s patient who was transplanted with his own hiPSC-DAN progenitors [7].

Many practical concerns arise with autologous transplantation. Autologous hiPSC-cell therapies are labour-intensive and a highly time-consuming process. The time pressure often associated with treating conditions such as severe SCI or stroke is incompatible with the time required to perform personalized stem cell work (e.g., derivation of hiPSC from the patient, cell banking, and differentiation into the relevant cell type). Early neuronal transplantation following SCI is indeed very beneficial as it can potentially avoid secondary injury and enhances recovery [48,49]. The use of allogeneic hiPSC/hESC lines for subsequent administration into SCI patients is therefore likely more feasible. Another concern is the disease status of the donor cells from patients who have disease-specific genetic backgrounds. To avoid disease recurrence, the disease-causing mutation would need to be corrected in hPSC prior to transplantation, or allogeneic transplantation may also become the preferred option for genetic degenerative diseases.

In contrast to hiPSC, an allogenic graft using hESC would incontestably require some form of pharmacological immunosuppression to avoid rejection by the recipient’s immune system. Long term immunosuppression comes with risks of infections and cancer and may lead to serious complications such as cardiovascular diseases [165]. The highly polymorphic major histocompatibility complex (MHC), known as HLA (Human Leukocyte Antigen) Class I and II in human, allows the immune system to recognize the body’s own cells and target foreign elements. HESCs express low level of HLA class I but not class II molecules. It has first been suggested that hESCs possess unique immune-privileged characteristics as they have a considerably weaker capacity to stimulate T cells compared with other graft types and can evade immune allo-rejection [166,167]. However, allogeneic NK cells can eliminate human ESCs in vitro and in vivo [137,168]. Secondly, following transplantation hESC will undergo differentiation into various cell types that express HLA molecules, leading to robust T-dependent allogeneic rejection.

In organ transplantation, matching HLA-A, -B, and -DR, improves the graft survival rates [169] and could represent a promising approach to reduce the use of immunosuppressant drugs in hPSC transplantation procedures. However, matching HLA types between donors and recipients is notoriously difficult. One way to produce HLA matched cells for cell-based therapy is by using histocompatible hPSC with homozygous HLA loci. This strategy not only significantly reduces the possibility of the derived cells being rejected by the recipient’s immune system but also makes a single cell line suitable for millions of patients. A relatively small number of HLA homozygous lines could be sufficient to provide immune matched cells to a large percentage of the world’s population. It is estimated that by selecting those homozygous for the 10 most frequent HLA-A,- B, or -*DRB1* haplotypes, 10, 75, and 140 HLA-homozygous iPSC lines would match approximately 50%, 80%, and 90% of the Korean/Japanese population, respectively [170,171]. This strategy would be more challenging for populations with higher racial and genetic diversity such as the US. A bank of 100 hiPSC lines homozygous for the most frequent HLA in each population could match up to 88% of European Americans, 63% of Asian Americans, 52% of Hispanic Americans and 45% only of African American [172]. Allele-specific HLA targeting was also performed in heterozygous hiPSC to generate pseudo-homozygous HLA hiPSC that could reduce the number of donors needed for HLA matching [173]. MHC matching was tested for dopaminergic transplants in NHPs and was able to reduce the immune response and improve engraftment of the transplant [174]. This idea has however been recently challenged. In the absence of immunosuppression, MHC matching alone is insufficient to prevent long-term rejection of iPSC-derived neuronal grafts in the lesioned brain of NHP [175]. Similarly, although the immunogenicity of allogeneic iPSC-cardiomyocytes (CMs) was reduced by MHC-matching, it was not completely abolished and an appropriate level of immunosuppression was required for successful engraftment in the heart of macaques [176].

As an alternative, a number of groups have set out to generate universal stem cells capable of evading any patient’s immune system. These approaches consist of manipulating genes that encode for HLA I and II proteins [173,177,178,179]. While ablation of HLA class II can be achieved by targeting its transcriptional master regulator CIITA, disruption of the *Beta-2Microglobulin* (*B2M*) gene eliminates surface expression of all HLA class Ia and b molecules and prevent the cells from being recognized as allogeneic by CD8+ T cells. However, *B2M* KO hPSC become vulnerable to NK cell-mediated rejection through missing-self response. Forced expression of minimally polymorphic HLA-E is one way to confer resistance to NK cells when HLA I is removed [180]. Individual deletion of the highly polymorphic HLA class Ia (HLA-A,-B,-C) genes represent another strategy to protect the donor cells from CD8^+^ T cell-mediated cytotoxicity without losing the HLA class Ib (non-polymorphic HLA-E,-G) protective function against NK cells mediated lysis [173,179]. Over-expression of *PDL1* and *CD47* also confers an additional advantage by protecting cells from T cell rejection and preventing macrophage engulfment respectively [177,179]. Engineered hypo-immunogenic hPSC showed reduced immune rejection in vivo*,* unlocking their full potential for regenerative medicine.

### 3.2. Regeneration of the Central Nervous System

In humans, or in mammals in general, the adult CNS has a limited regenerative capacity and is unable to fully restore its function after injury. Consequently, CNS trauma generally results in severe and persistent functional deficits which drastically reduce quality of life. This regeneration failure is mainly due to the very limited number of neural stem cells that the CNS retains into adulthood, which prevents the replacement of lost neurons, and to its low capacity to repair damaged neurons. Human brains, in particular, are controversially reported to have limited or no adult de novo neurogenesis [181,182,183]. The adult CNS also shows limited axonal regeneration which results from the lack of intrinsic growth capability of existing neurons (due to insufficient growth promoting signals) and an inhibitory CNS microenvironment.

#### 3.2.1. CNS Microenvironment

The non-permissive nature of the CNS environment for axonal regeneration is largely attributable to non-neuronal components of the CNS [184], in particular the neurite growth inhibitory myelin proteins secreted by oligodendrocytes.

Lesions in the CNS cause changes in the microenvironment directly surrounding the site of the injury. Myelin, a lipid-rich substance normally insulating the axons, becomes severely damaged and leaves the axons exposed and vulnerable to myelin-derived inhibitory molecules such as Nogo, myelin-associated glycoprotein (MAG), and oligodendrocyte-myelin glycoprotein (OMgp). Nogo-A is considered to be the major inhibitor of axonal regeneration and is thought to launch a signaling cascade which induces cytoskeletal changes and destabilization in the growth cones [185]. Its expression varies greatly after SCI, initially decreasing the first 3 days to reach a peak after 7 days. High levels of Nogo-A may be required for its inhibitory action. The fluctuation in expression of Nogo-A suggests a 7-day window for treatment of SCI [186,187]. Current approaches have focused on administration of Nogo-receptor blocking peptides and anti-NogoA blocking antibodies to promote axon regeneration in rodent and primate models of SCI. Neutralization of the neurite growth inhibitor Nogo-A by intrathecal antibodies has shown enhanced functional recovery and regeneration of injured CNS in SCI rats [188].

Another critical component of the limited regenerative potential of the adult CNS is neuro-inflammation. In addition to severed axons, trauma to the spinal cord causes blood-spinal cord barrier disruption, which in turn causes localized neuronal death. This induces further oxidative stress and glutamate release causing excitotoxic death of neighboring neurons and glia. Shortly after the injury, resident microglia become reactivated, initiating a robust immune response by secreting various cytokines, and peripheral macrophages are recruited to the lesion site. This inflammatory response may have additional deleterious effect on neuronal regeneration and prevent functional recovery. Depletion of peripheral macrophages [189] and administration of the anti-inflammatory drug minocycline [190] have been shown to enhance axonal regeneration and improve functional recovery after SCI. However, a beneficial role of neuro-inflammation has also been described. Macrophage/microglia activation, by intraspinal injection of pro-inflammatory molecules, results in a better regenerative outcome after SCI, either by promoting sectioned axons regrowth [191] or reducing axonal loss [192]. These conflicting results have led to considerable debate concerning the neurotoxic or neuroprotective effect of inflammation in CNS regeneration.

Because of the importance of the immune component in CNS regeneration, caution is necessary in translating preclinical models of neurodegenerative diseases to human. As discussed previously, functional recovery following transplantation of various hES/hiPS cell-derived neural cell types has been well reported in rodents with SCI, PD, stroke or MS. However, in most cases, transplantations were tested in immune-deficient animals and cannot translate directly to human patients. Interestingly, unlike successful studies performed in immuno-deficient mice, the transplantation of hiPSC-NPCs in immuno-competent SCI mice treated with tacrolimus (an immunosuppressive drug) led to very disappointing results. Behavioral assessment showed failure to improve functional recovery and overall poor long-term survival [43]. Whether the discrepancy in the results can be attributed to the difference in immunocompetency of the mouse models is unclear but would benefit from further investigation. Similarly, chronic neuropathic pain cell therapy studies used immunosuppressed animals [50,51] which may affect the results. Whilst transplantation studies using syngenic fetal mouse material suggest this will not affect analgesia, given the important role of immune cells in pain pathogenesis [193,194] or CNS regeneration, further investigation is needed to establish the potential effect of immunosuppression.

#### 3.2.2. Glial Scarring

The glial scar is one of the most established barriers of CNS regeneration. The glial scar has been largely studied in the context of SCI, but also occurs after traumatic brain injury or ischemic stroke and in neurodegenerative diseases such as AD, or in demyelinating and inflammatory pathologies such as MS.

As discussed previously, damage to the spinal cord is followed by an acute inflammatory response. Resident microglia are activated, and peripheral immune cells (including macrophages and lymphocytes) enter the lesion site. This inflammatory reaction eventually becomes chronic and at this point, astrocytes proliferate, hypertrophy and overlap to contain the damage and isolate the lesion from the spared tissue, forming a regeneration-inhibiting glial scar. The repressive nature of the glial scar has been largely attributed to a high concentration of ECM (extra-cellular matrix) and inhibitory proteins, including myelin-associated chondroitin sulfate proteoglycans (CSPGs) secreted by astrocytes [195]. CPSGs have been described to impair neuronal growth in vitro by signaling through the Rho/ROCK pathway [196]. Degradation of CSPGs using chondriotinase enzyme was sufficient to restore synaptic activity below the lesion site in SCI rats [197] and promoted functional recovery following SCI [198,199]. Similarly, an increase of CSPG concentration caused a decrease in neurite length in vitro [200]. Other cell types implicated in glial scarring include pericytes. The inhibition of pericytes proliferation following SCI was recently shown to enhance neuronal survival, cause a reduction in glial scar formation and improve functional recovery after SCI [201].

Conversely, a beneficial role of the glial scar has also been described. By preventing resident astrocytes from proliferating after SCI (using conditional genetic ablation), recovery from spinal cord lesions was reduced. The injured area in the spinal cord expanded, more axons were severed and an increased neuronal death was observed in mice unable to generate glial scars. This study also demonstrated that astrocytes of the glial scar are a major source of neurotrophic factors (CNTF, HGF, IGF1) required for the survival of neurons adjacent to the lesion after SCI [202]. Furthermore, a supportive role of the glial scar in regeneration of neurons has also recently been identified. Preventing astrocytic scar formation, by selectively killing proliferative scar-forming astrocytes or deleting of STAT3 signaling specifically in astrocytes, stopped stimulated axon regrowth in mice. Prevention of the scarring does not reduce overall CSPG production post-injury, due to the quantity of non-astrocytic cells producing CSPGs [203]. The astrocytic glial scar has also been recently shown to not prevent remyelination in a rat model of MS [204]. These results demonstrate the importance of apparently growth inhibiting programs in reacting to CNS damage appropriately, and highlight a tightly regulated mechanism that leads to successful repair.

## 4. Conclusions

Over the past decades, neurodegenerative diseases and nervous system injury have been a major focus of regenerative medicine, with many studies dedicated to developing efficient and clinically relevant hPSC replacement therapies for the treatment of a variety of neurological disorders. However, the clinical feasibility of these therapies requires further assessment. A deeper understanding of differentiation pathways and mechanisms will permit the development of defined cell populations that can be used as more potent therapeutics. Moreover, the increased risk of cancer caused by the use of hiPSCs raised serious reservations regarding the development of autologous cell therapies. Future directions will concentrate on banking clinically safe and universally compatible hPSC to overcome the challenge of immune rejection. Nonetheless, hPSC therapies provide genuine hope for a wide range of currently devastating degenerative diseases, and will eventually change the way we see aging and the associated tissue degeneration by redefining the impossible.

## Figures and Tables

**Table 1:** Overview of hPSC-based therapy studies performed in neurodegenerative diseases animal models.

Disease	Cell Type Transplanted	Transplant Site	Animal Model	Readout	Reference
PD	hESC-DANs	Striatum	Rat, 6-OHDA	apomorphine-induced rotations, adjusting step test, cylinder test.	[13]
	hESC-DANs	Striatum in mice, putamen in monkeys	Mouse Monkeys, MPTP	MRI, Neurological rating scale, video-based analysis of spontaneous movements	[14]
	hESC-midbrain DANs	Striatum in mice and rats, Putamen in monkeys	Mouse, 6-OHDA Rats, 6-OHDA Monkeys, MPTP	Amphetamine-induced rotations (Rats and mice), stepping test (rats), cylinder test (rats).	[15]
	hESC-midbrain DANs	Striatum	Rat, 6-OHDA	Amphetamine-induced rotation Cylinder test	[16]
	hESC-midbrain DANs	Striatum	Rat, 6-OHDA	MRI MR spectroscopy PET-Scan Amphetamine-induced rotations	[17]
	hESC-DA progenitor cells	Striatum	Rat, 6-OHDA Mouse, 6-OHDA	Amphetamine-induced rotations Cylinder test	[18]
	hiPSC-DANs	Striatum	Rat, 6-OHDA	Amphetamine- and apomorphine-induced rotations	[19]
	hiPSC-DANs	Striatum	Rat, 6-OHDA Mouse, α-Synuclein Tg	Amphetamine-induced rotations	[20]
	hiPSC-NPCs and hiPSC-DANs	Striatum	Rat, 6-OHDA	Amphetamine-induced rotations	[21]
	hiPSC-NSCs	Striatum	Rat, 6-OHDA	Turning-over test Rotation-rod test	[22]
	hiPSC-NPCs	Putamen	Monkey, MPTP	Raisin pick up test Neurological rating scale	[23]
	hiPSC-DA progenitor cells	Putamen	Monkey, MPTP	Neurological rating scale, Video-based analysis of spontaneous movements	[24]
Stroke	hiPSC-lt-NES cells	Striatum, Cortex	Rat & Mouse, MCAo	Staircase and corridor tests	[25]
	hiPSC-cortical fated lt-NES	Cortex	Rat, MCAo	Immunoelectron microscopy Rabies virus retrograde synaptic tracing electrophysiology	[26]
	hiPSC-cortical fated lt-NES	Cortex	Rat, MCAo	Cylinder and stepping test	[27]
	hiPSC-cortical fated lt-NES	Cortex	Rat, MCAo	Rabies virus retrograde synaptic tracing Immunoelectron microscopy Optogenetics Electrophysiology Cylinder test	[28]
	hiPSC-NPCs	Striatum	Mouse, MCAo	Modified neurological severity score (mNSS)	[29]
	hiPSC-NPCs	Penumbra region of the cortex	Mouse, MCAo	Adhesive removal test – latency and removal time	[30]
	hiPSC-NPCs	Striatum	Rat, MCAo	Rotarod test Stepping test mNSS Apomorphine-induced rotation tests	[31]
	hiPS-NPCs	Right cortex	Rat, Incision in common carotid artery	Vibrissae-elicited forelimb placing test Cylinder test	[32]
	hiPSC-NSC	Cortex surrounding lesion	Pig, MCAo	MRI and histology (no functional measurement)	[33]
Epilepsy	hESC-MGE progenitors	Hippocampus	Mouse, Pilocarpine-induced TLE	EEG recording Novel object recognition test Locomotion test Handling test	[34]
	hESC-MGE progenitors	Hippocampus	Mouse, Pilocarpine-induced TLE	Morris Water Maze test, EEG recording	[35]
Learning and Memory/AD	hiPSC-NPCs (with cholinergic neuronal phenotype)	Bilateral hippocampus	Mouse, Tg PDAPP	Morris Water Maze test	[36]
	hESC-BFCN Progenitors	Bilateral Hippocampus	Mouse, Tg 5XFAD and	Morris Water Maze test Electrophysiology (Whole-Cell patch-clamp)	[37]
	hiPSC-ML/NEP2	Hippocampus	Mouse, Tg 5XFAD	Immuno-histochemistry (no functional assay)	[38]
Multiple Sclerosis	hiPSC-OPCs	Corpus Callosum	Mouse, Shiverer/rag2	Survival time	[39]
	hiPSC-OPCs	Forebrain	Mouse, Shiverer/rag2	Immuno-histochemistry (no functional assay)	[40]
Spinal Cord Injury	hiPSC-NSCs	Lesion epicentre	Mouse, moderate contusive SCI at T10 level	Rotarod test BMS score DigiGait system	[41]
	hiPSC-lt-NES	Lesion epicentre	Mouse, contusive SCI at T9 level	BMS locomotor scale	[42]
	hiPSC-NPCs	Lesion epicentre	Mouse, moderate contusive SCI at T11 level	BMS scale CatWalk-automated gait analysis	[43]
	hiPSC-NPCs	Lesion epicentre at T11	Mouse, and compression injury T11	Open-field, footprint analysis	[44]
	hiPSC-NSCs	Lesion epicenter at C5	Rat, C5 lateral hemisection lesions	Grid-walking Grooming Vertical exploration (no functional improvement)	[45]
	hiPSC-NPCs	Lesion epicenter at T8	Rat, balloon-induced compression lesion at T8 level	BBB test Beam walking test Rotarod test Plantar test	[46]
	hiPSC-NSCs	Lesion epicenter	Marmoset, moderate contusive SCI by weight-drop at C5 level	Open field rating scale Bar grip test Cage climbing test	[47]
	hESC-OPCs	Lesion epicenter at T8 of spinal cord.	Rat, contusive injury by weight-drop at T8 level	SSEP (Somatosensory Evoked Potentials) evaluation	[48]
	hiPSC-OPCs	T8 of spinal cord.	Rat, moderate contusive SCI by weight-drop at T8 level	BBB locomotor rating scale	[49]
	hESC-MGE (GABAergic progenitors)	Lumbar enlargement level L3–L5.	Mouse, moderate contusive SCI at T13 level	BMS scale, Open field, Von Frey, Over-grooming, Assessment of bladder function by analysis of voluntary Voiding Pattern and Cystometry	[50]
Neuropathic Pain	hESC-MGE progenitors	Spinal Cord, Lumbar enlargement (L3–L5)	Mouse, moderate contusive SCI at T13 level	BMS scale, Open Field, Von Frey, Over-grooming.	[50]
	hiPSC-GABAergic neurons	Spinal Cord, Lumbar enlargement L1.	Mouse, SNI	BMS scale Von Frey, Acetone Open Field,	[51]

PD: Parkinson’s Disease; AD: Alzheimer’s Disease. SCI: Spinal Cord injury; hESC: human Embryonic Stem Cells; NSC: Neural Stem Cells; NPC: Neuronal Progenitor Cells; hiPSC: human Pluripotent Stem Cells; DANs: Dopaminergic neurons; lt-NES: long-term self-renewing Neuro-Epithelial-like Stem; ML/NEP2: Macrophage-Like/Neprilysin-2; BFCN: Basic Forebrain Cholinergic Neurons; OPC: Oligodendrocyte Progenitor Cells; MGE: Medial Ganglionic Eminence; SNI: Spared Nerve Injury.

**Table 2:** Clinical trials using hPSC derivatives for diseases/injuries of the nervous system.

Disease	Treatment Type	Phase	Clinical Trial Identifier	Country
PD	parthenogenetic hESC-NSC (ISC-hpNSC)	Phase I	NCT02452723	Australia
	HLA-matched hESC-NPC	Phase I/II	NCT03119636	China
	hiPSC-DA Progenitors	Phase I/II	JMA-IIA00384 UMIN000033564	Japan
Amyotrophic Lateral Sclerosis (ALS)	hESC-Astrocystes (AstroRx)	Phase I/II	NCT03482050	Israel
SCI	hESC-OPC (AST-OPC1)	Phase I/II	NCT02302157	USA

PD: Parkinson’s Disease; SCI: Spinal Cord injury; hESC: human Embryonic Stem Cells; HLA: Human Leucocyte Antigen; NSC: Neural Stem Cells; NPC: Neuronal Progenitor Cells; hiPSC: human Pluripotent Stem Cells; DA: Dopamine; OPC: Oligodendrocyte Progenitor Cells.
